# Effect of cAMP derivates on assembly and maintenance of tight junctions in human umbilical vein endothelial cells

**DOI:** 10.1186/1471-2121-11-68

**Published:** 2010-09-07

**Authors:** Michaela Beese, Kristin Wyss, Marion Haubitz, Torsten Kirsch

**Affiliations:** 1 Division of Nephrology, Department of Internal Medicine, Hannover Medical School, Carl-Neuberg-Str. 1, 30625 Hannover, Germany

## Abstract

**Background:**

Endothelial tight and adherens junctions control a variety of physiological processes like adhesion, paracellular transport of solutes or trafficking of activated leukocytes. Formation and maintenance of endothelial junctions largely depend on the microenvironment of the specific vascular bed and on interactions of the endothelium with adjacent cell types. Consequently, primary cultures of endothelial cells often lose their specific junctional pattern and fail to establish tight monolayer *in vitro*. This is also true for endothelial cells isolated from the vein of human umbilical cords (HUVEC) which are widely used as model for endothelial cell-related studies.

**Results:**

We here compared the effect of cyclic 3'-5'-adenosine monophosphate (cAMP) and its derivates on formation and stabilization of tight junctions and on alterations in paracellular permeability in HUVEC. We demonstrated by light and confocal laser microscopy that for shorter time periods the sodium salt of 8-bromoadenosine-cAMP (8-Br-cAMP/Na) and for longer incubation periods 8-(4-chlorophenylthio)-cAMP (pCPT-cAMP) exerted the greatest effects of all compounds tested here on formation of continuous tight junction strands in HUVEC. We further demonstrated that although all compounds induced protein kinase A-dependent expression of the tight junction proteins claudin-5 and occludin only pCPT-cAMP slightly enhanced paracellular barrier functions. Moreover, we showed that pCPT-cAMP and 8-Br-cAMP/Na induced expression and membrane translocation of tricellulin.

**Conclusions:**

pCPT-cAMP and, to a lesser extend, 8-Br-cAMP/Na improved formation of continuous tight junction strands and decreased paracellular permeability in primary HUVEC. We concluded that under these conditions HUVEC represent a feasible *in vitro *model to study formation and disassembly of endothelial tight junctions and to characterize tight junction-associated proteins

## Background

Endothelial cells line the surface of all vascular or lymphatic vessels and are connected by intercellular junctions consisting of tight junctions (*zonula occludens*), adherens junctions and gap junctions [1]. These junctional complexes control a variety of cellular mechanisms like adhesion, paracellular transport or signaling events [2]. In contrast to most epithelial cells that are characterized by a highly regulated organization of cellular junctions interendothelial junctions are far from being static structures. In fact, both adherens junctions and tight junctions seem to be intermingled throughout the cell-cell contact areas [3]. Tight junctions consist of several transmembrane or membrane-associated proteins including the membrane spanning claudins (CLDN), occludin (OCLN) and junctional adhesion molecules (JAM) [3]. Tricellulin (TRIC) represents another tight junction-associated integral membrane protein that is localized at tricellular junctions in epithelial cells and is described to regulate paracellular permeability [4,5].

These proteins are connected to the actin cytoskeleton via adaptor proteins like the *zonula occludens *(ZO) proteins ZO-1, ZO-2, ZO-3, cingulin, AF6 or 7H6 [6-9]. The main constituents of endothelial adherens junctions are vascular endothelial cadherin (VE-cadherin) and catenins that link adherens junctions to the cytoskeleton [10]. Recently, a study published by Taddei and co-workers demonstrated the importance of adherens junctions in controlling proper tight junction formation in endothelial cells *in vitro *[11].

A common problem of working with primary endothelial cells is the rapid loss of many of their endothelial characteristics as soon as they are cultivated *in vitro*. This de-differentiation results from the missing interaction between endothelial cells and the specific microenvironment that is necessary for endothelial specifity [12]. As a consequence, the use of primary endothelial cells for studying endothelial junctions comprises some limitations, e.g. fragmentary assembly of tight junctions, increased paracellular permeability or low transelectrical resistance. Therefore, many efforts have been done to overcome these limitations. For instance, co-cultures of microvascular endothelial cells and vascular smooth muscle cells, pericytes or astrocytes, or conditioned supernatant of these cells, proved useful in restoring endothelial barrier properties [13-15]. Also activation of cyclic 3',5'-adenosine monophosphate (cAMP)-dependent protein kinase (PKA) by cAMP or addition of dexamethasone or hydrocortisone have been shown to successfully improve endothelial junction architecture [16-19].

A convenient and easy to obtain source for primary human endothelial cells are umbilical cords, and human umbilical vein endothelial cells (HUVEC) are widely used for studying different endothelial cell-related questions [20-22]. Although HUVEC do not possess the specific barrier properties found in highly impermeable microvascular beds like the blood-brain barrier, they are suitable for studying architecture and formation of endothelial intercellular junctions. Nevertheless, it is necessary to induce formation of intercellular junctions in HUVEC as these cells, even when grown in tight monolayer, display intercellular gaps and discontinuous junction strands.

The aim of the present study was to compare and validate different cAMP analogues regarding their effect on expression of junction-associated proteins and on formation of continuous tight junction strands as well as on paracellular permeability in freshly isolated HUVEC.

## Results

### Morphology and cellular integrity of HUVEC stimulated with different cAMP derivates

**Figure 1:**
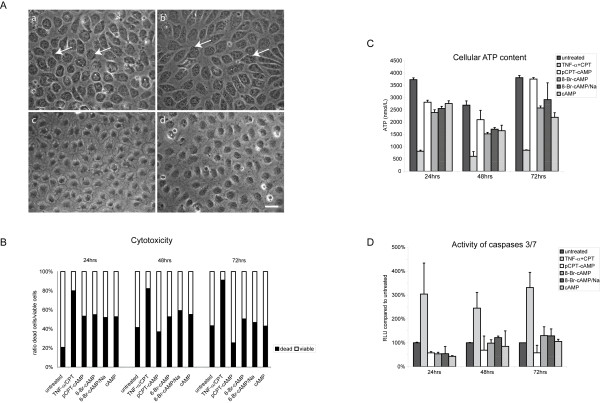
**Morphology and cellular integrity of HUVEC treated with different cAMP derivates**. (A) Stimulation of HUVEC for 72 h with cAMP or 8-Br-cAMP induced a regular monolayer but led to extensive vacuolization (white arrows) that may represent signs of cytotoxicity (a,b). Cells stimulated with 8-Br-cAMP/Na or pCPT-cAMP grew in a dense monolayer with regularly arranged cells and visible cell borders (c,d) without any signs of cytotoxicity. Shown are representative micrographs of at least three experiments with cells derived from different donors. (B) All four cAMP derivates induced enhanced cellular cytotoxicity in the first 24 h whereas after 72 h the ratio of dead and viable cells is comparable or, for pCPT-cAMP, even below that of untreated cells. TNF-α/CPT treated HUVEC served as positive control. (n = 3) (C) Exposure to cAMP, 8-Br-cAMP or 8-Br-cAMP/Na induced a slight reduction in intracellular ATP level over the whole time period. Treatment with pCPT-cAMP resulted in a partial decrease of cellular ATP concentrations after 24 h but showed no differences to untreated cells after 72 h. (n = 3) (D) All four cAMP derivates induced a slight decrease in caspase3/7 activity after 24 h. After 48 h and 72 h level of activated caspases are comparable to those of untreated cells (n = 4). Scale bar represent 25 μm.

HUVEC cultivated for several days in EGM-2 medium formed a monolayer with endothelial-like cobblestone morphology. Nevertheless, the cells differed in size and shape and the arrangement of the cells occurred rather disorganized. Addition of cAMP or its derivates greatly improved the formation of dense monolayer and the endothelial cells showed much more uniformity in size and shape (Figure 1A). Unexpectedly, cAMP and the bromine derivate 8-Br-cAMP seemed to exhibit cytotoxic side effects as HUVEC exposed to these compounds for 72 h showed a dense ring of vacuoles around the nucleus and the cell borders seemed to vanish. In contrast, HUVEC treated with pCPT-cAMP or 8-Br-cAMP/Na for 72 h showed no sign of cytotoxicity and the cell borders were clearly visible in phase contrast microscopy (Figure 1A). In order to analyze the effect of the different cAMP derivates on cellular homeostasis we determined the ratio of viable and dead cells by measuring the release of a cell membrane-impermeable protease. HUVEC treated with a combination of tumor necrosis factor-alpha (TNF-α, 3 nmol/l) and camptothecin (CPT, 0.15 μmol/l) served as positive control. All four cAMP derivates showed a higher ratio of dead cells compared to untreated cells in the first 24 h. Nevertheless, this potential cytotoxic effect disappeared in the next 24 h and after 72 h the ratio of dead cells was comparable to or, in the case of pCPT-cAMP, even below that of untreated cells (Figure 1B). Concordant with these results intracellular concentrations of ATP as indicator for metabolic activity slightly decreased in the first 24 h upon exposure to the cAMP derivates compared to untreated cells. After 72 h pCPT-cAMP-treated and untreated cells showed equivalent levels of intracellular ATP whereas the other three derivates induced a slight decrease of cellular ATP (Figure 1C). We also determined activation of caspases 3 and 7 as indicator for induction of apoptosis. Activation of caspases 3/7 did not significantly differ upon treatment with the cAMP derivates compared to untreated cells whereas stimulation with TNF-α and CPT strongly increased the signals for active caspases 3/7 (Figure 1D).

### Cellular localization and distribution of junction-associated proteins

**Figure 2:**
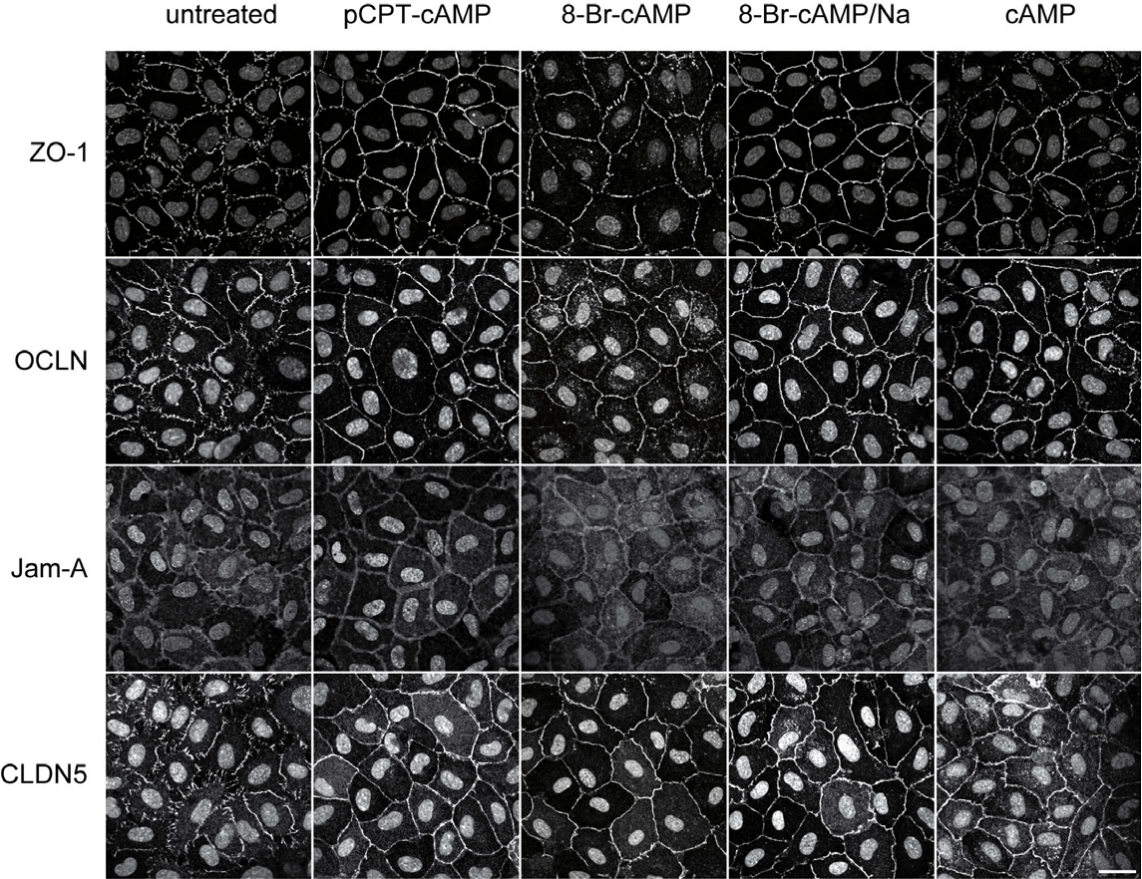
**Short-term effect of cAMP and its derivates on formation of tight junctions**. Cells were stimulated for 24 h with different cAMP derivates, stained for tight junction-associated proteins ZO-1, OCLN, CLDN5 and Jam-A and analyzed by confocal laser microscopy. The nucleus was counterstained with DAPI. Depicted are representative micrographs of at least three independent experiments using HUVEC from different donors. Scale bar represents 25 μm.

**Figure 3:**
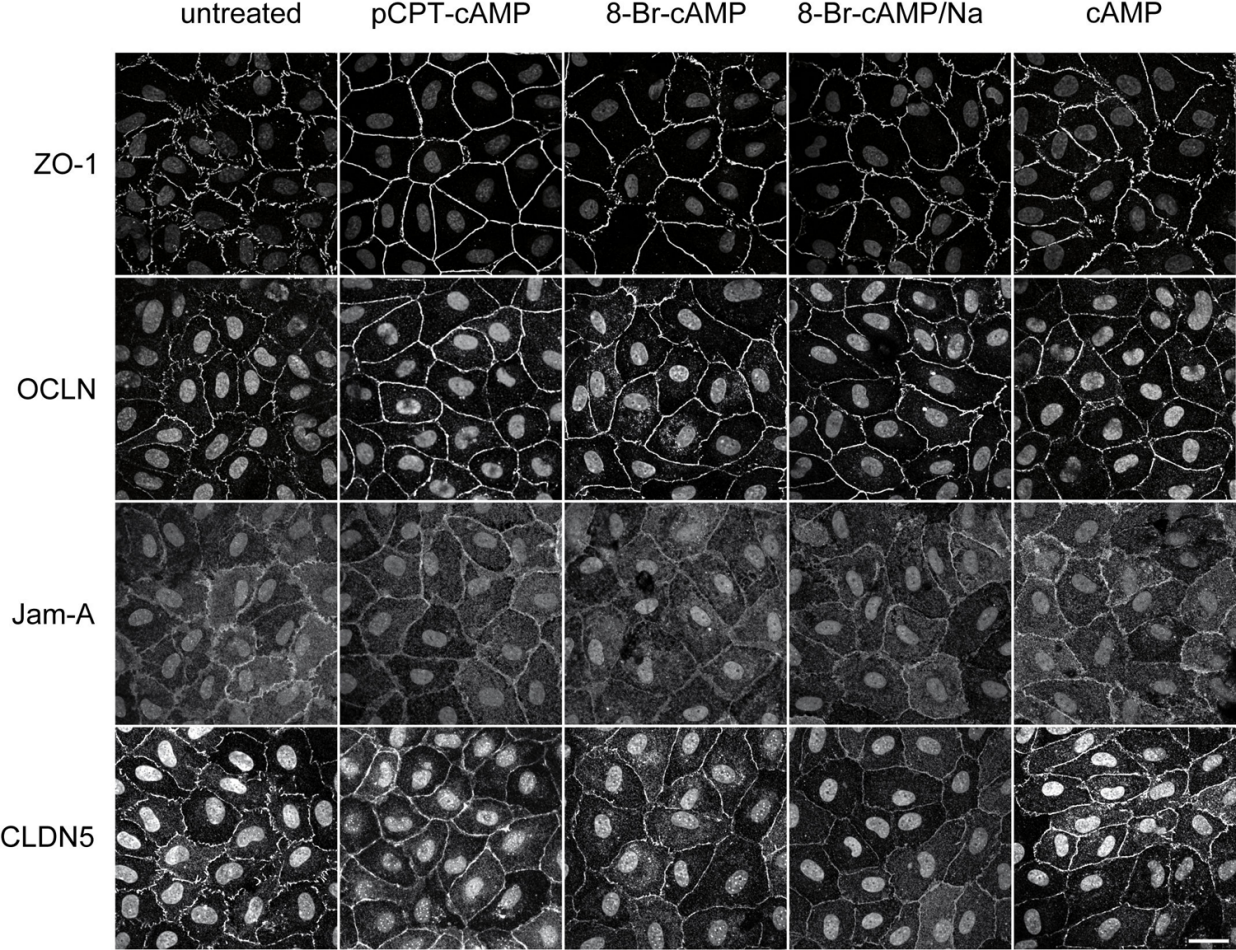
**Long-term effect of cAMP and its derivates on formation of tight junctions**. HUVEC were stimulated for 72 h with the different cAMP derivates, stained for tight junction-associated proteins ZO-1, OCLN, CLDN5 and Jam-A and analyzed by confocal laser microscopy. The nucleus was counterstained with DAPI. Shown are representative micrographs of at least three independent experiments using HUVEC from different donors. Scale bar represents 25 μm.

We next performed immunostainings for the adherens junction protein VE-cadherin and the tight junction associated proteins ZO-1, OCLN, Jam-A and CLDN5 to analyze their cellular distribution in response to cAMP or its derivates. HUVEC cultivated for 24 h in EGM-2 medium without any additional supplementation with cAMP or its derivates showed discontinuous VE-cadherin-positive strands with intercellular gaps. In the next 48 h these intercellular gaps disappeared but the discontinuity of the VE-cadherin-positive strands remained (additional file 1). Likewise, the staining pattern for the tight junction-associated proteins ZO-1, OCLN, CLDN5 and Jam-A revealed discontinuous or intermittent junction strands suggesting that EGM-2 medium alone is not sufficient to induce proper assembly of continuous tight junction strands (Figure 2 and 3). In contrast, addition of cAMP for 24 h induced the formation of continuous junction strands as shown by immunostainings for ZO-1, OCLN, CLDN5 and Jam-A (Figure 2). Nonetheless, we observed that the junction strands seemed to disintegrate again after stimulation periods for 72 h (Figure 3). Concordant with these results, HUVEC stimulated with 8-Br-cAMP showed continuous assembly of tight junction strands in the first 24 h but after 72 h the junction strands disassembled again. The same temporal pattern could be observed after stimulation with 8-Br-cAMP/Na. Although this compound obviously showed no cytotoxicity the most notably effect on inducing continuous adherens and tight junction-strands occurred in the first 24 h whereas in the next 48 h the junctions started to separate again (Figure 2 and 3). These results suggest that 8-Br-cAMP/Na is preferentially applicable for short term experiments. pCPT-cAMP, on the other hand, showed its greatest effects on HUVEC after incubation for 72 h (Figure 3). After this time period immunostainings for ZO-1, OCLN, Jam-A or CLDN5 revealed continuous tight junction strands. The cells displayed a regular shape and there was no sign of cytotoxicity. The appropriate stainings of ZO-1, OCLN, Jam-A and CLDN5 after stimulation with cAMP or its derivates for 48 h are shown in additional file 2. To further analyze the effect of pCPT-cAMP on survival and cytotoxicity we cultivated the cells for more than 10 days with pCPT-cAMP. Even after this time period the cells looked healthy and still grew in a tight monolayer (data not shown).

### cAMP derivates influence formation and dissociation of tight junctions in a calcium switch model

**Figure 4:**
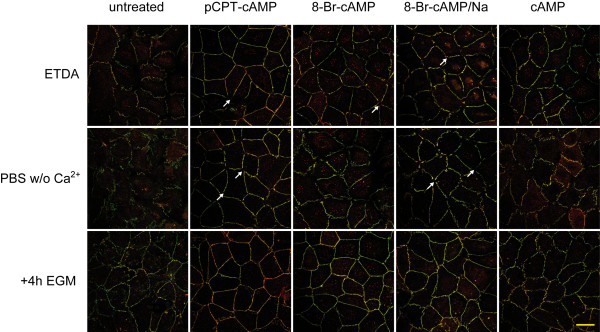
**cAMP derivates influence formation and dissociation of tight junctions in a calcium switch model**. HUVEC were treated for 24 h with cAMP or its derivates. Calcium was either chelated by addition of 3 mM EDTA or medium was exchanged with calcium-free PBS. After 2 h cells were washed and cultivated for additional 4 h in EGM medium supplemented with the appropriate cAMP derivates. Cells were fixed and stained for CLDN5 (red) and ZO-1 (green). White arrows indicate specific loss of tricellular junctions. Shown are representative micrographs of three independent experiments. Scale bar represents 25 μm.

We then performed calcium switch experiments to analyze whether the different cAMP derivates modulate the responsiveness of HUVEC to altered calcium concentrations in the culture medium. In a first set of experiments HUVEC were stimulated with the different cAMP derivates for 24 h before they were cultivated under low calcium for 2 h. Addition of EDTA (3 mmol/l) to the normal culture medium or cultivation in calcium-free buffer resulted in the specific loss of tricellular junctions in HUVEC exposed to pCPT-cAMP or 8-Br-cAMP/Na. Subsequent reconstitution of the normal calcium concentration restored the continuous junction strands. Interestingly, removal of calcium induced a much greater effect on opening of tight junction in cells exposed to cAMP or 8-Br-cAMP. In these cells not only the tricellular junctions were lost but also bicellular junctions started to disintegrate. Restoring the original calcium concentration could reestablish continuous junction strands only in the 8-Br-cAMP-treated cells whereas cAMP-treated HUVEC failed to form continuous ZO-1 or CLDN5-positive strands. In untreated cells calcium depletion increased the discontinuity of junction strands, and restoring the original calcium concentration did not seem to have any beneficial effect on reorganization of cell-cell junctions (Figure 4). In a second approach the cells were treated with the cAMP derivates for 72 h before removal of calcium. Similar to the first experiments HUVEC pretreated with pCPT-cAMP or 8-Br-cAMP showed loss of tricellular junctions at low calcium concentrations but reestablished continuous junction strands after restoring the original calcium concentration. 8-Br-cAMP/Na and cAMP treated cells showed enhanced loss of tri- and bicellular junctions and failed to reestablish continuous tight junctions after restoring calcium concentration (data not shown).

### Expression of tight junction-associated proteins

**Figure 5:**
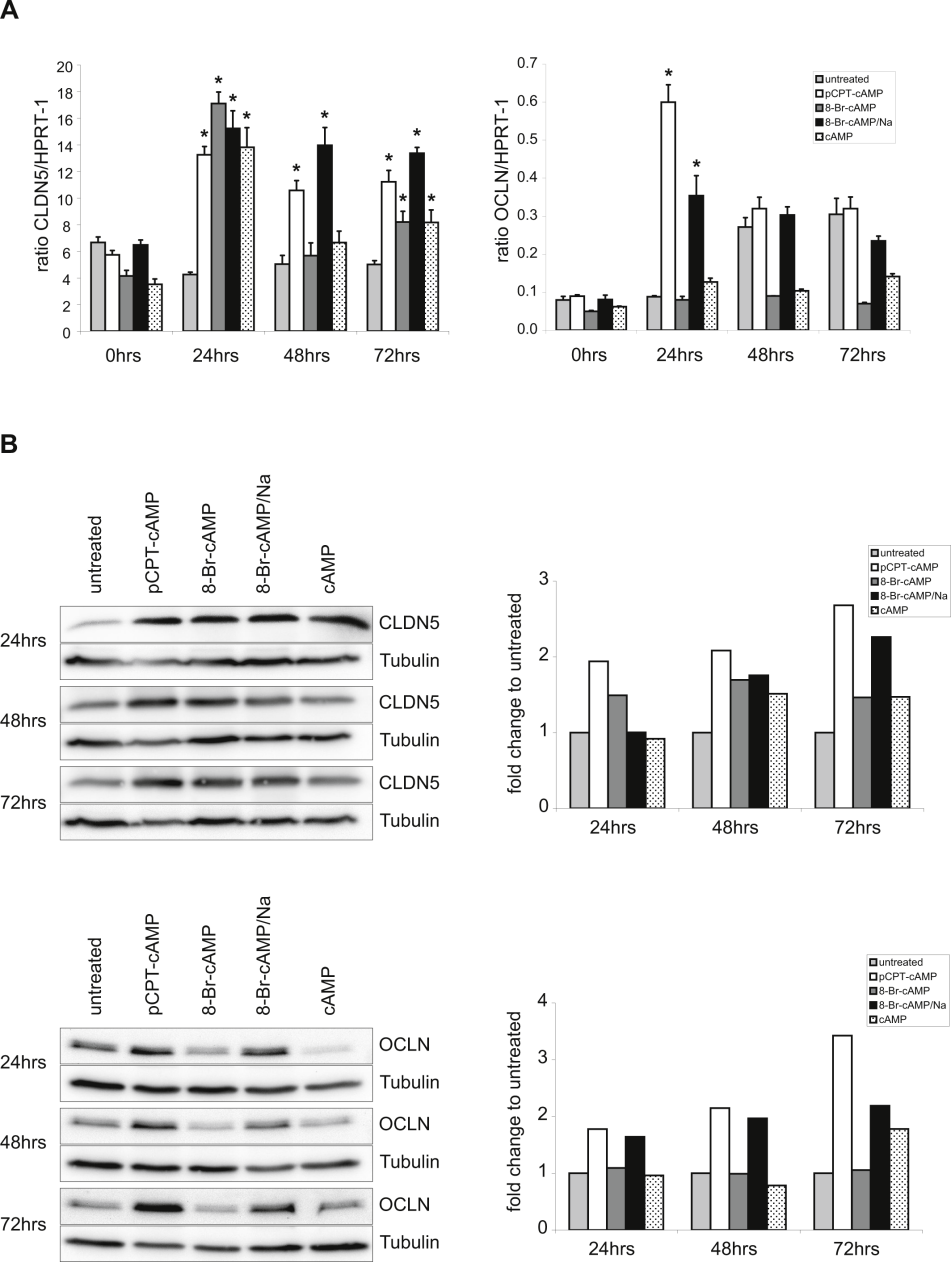
**cAMP and its analogues modulate mRNA and protein expression**. HUVEC were stimulated with cAMP or its derivates for up to 72 h and changes in transcript amount and protein expression were determined by real-time qPCR and western blotting. (A) real-time qPCR revealed a strong induction of CLDN5 and OCLN mRNA synthesis after stimulation with pCPT-cAMP and 8-Br-cAMP/Na over the whole time period whereas cAMP and 8-Br-cAMP induced only a temporal increase in CLDN5 transcript amount and obviously had no effect on OCLN mRNA expression. Untreated cells showed no differences in CLDN5 expression but a significant increase in OCLN transcript amount after 48 h. (n = 3). (B) Shown are western blots of HUVEC treated for 24 to 72 h with different cAMP derivates. Protein lysates of HUVEC were separated by electrophoresis, blotted and OCLN and CLDN5 were detected with specific antibodies. Protein amounts of tubulin served as loading controls. (n = 5); (*p < 0.01 compared to untreated controls).

We also tested the different compounds for their impact on gene expression and protein synthesis in HUVEC. Transcript amounts of ZO-1, Jam-A and VE-cadherin did not change significantly in treated cells compared to untreated controls (additional file 3). In contrast, cAMP and its derivates induced a significant increase in CLDN5 transcript amounts after 24 h. CLDN5 mRNA level in cells treated with pCPT-cAMP or 8-Br-cAMP/Na remained elevated whereas cAMP or 8-Br-cAMP in turn led to a decline in transcript expression after 48 h. CLDN5 transcript level in untreated cells remained almost unchanged over the whole time (Figure 5A). mRNA expression of OCLN was strongly enhanced after 24 h following treatment with 8-Br-cAMP/Na and pCPT-cAMP whereas untreated cells showed increased OCLN expression with a temporal delay of 24 h. Nevertheless, after 72 h the mRNA level did not differ significantly within these three groups. In contrast, stimulation with cAMP and 8-Br-cAMP did not induce any significant increase in OCLN transcript amount (Figure 5A). We then performed western blots to analyze whether the changes in gene expression had any influence on protein amounts. All cAMP derivates induced an increase in CLDN5 protein amount with pCPT-cAMP and 8-Br-cAMP/Na leading to the highest increase after 72 h. These results are consistent with the data obtained by real-time qPCR. Accordingly, HUVEC exposed to pCPT-cAMP and 8-Br-cAMP/Na displayed the highest level of OCLN protein (Figure 5B).

### Effects of cAMP derivates on tight junctions are partly dependent on PKA

**Figure 6:**
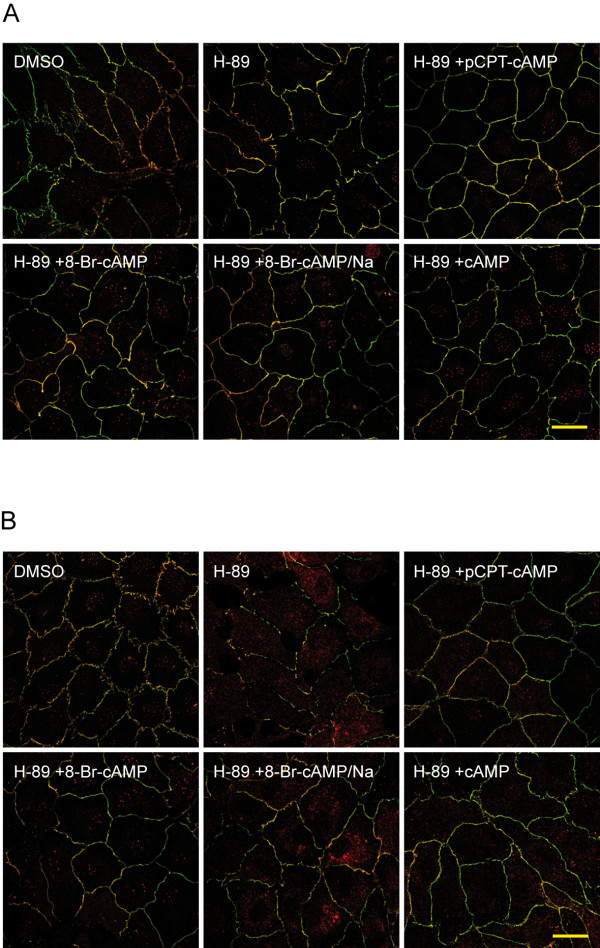
**cAMP-induced formation of junction strands is independent of PKA**. HUVEC were pretreated with H-89 and exposed to the different cAMP derivates for (A) 24 h or (B) 72 h. Cells were fixed and double stainings for ZO-1 (green) and CLDN5 (red) were performed. Shown are representative micrographs of three independent experiments. Scale bar represents 25 μm.

As evidence exists that cAMP signals through PKA-dependent and PKA-independent mechanisms we analyzed whether the observed effects of the cAMP derivates were modulated by PKA. HUVEC were pretreated with the PKA-specific inhibitor H-89 (10 μmol/l), subsequently exposed to the different cAMP derivates for up to 72 h and mRNA and protein amount of OCLN and CLDN5 were measured by real-time qPCR and western blotting, respectively. Inhibition of PKA by H-89 clearly prevented the increase in transcript and protein synthesis induced by cAMP or its derivates (additional file 4). We then performed immunostaining for ZO-1 and CLDN5 in HUVEC pretreated with H-89 and exposed to the different cAMP derivates for 24 and 72 h. As could be seen in Figure 6A, stimulation with the PKA inhibitor alone seemed to induce swelling of the cells. This effect might be explained by increased influx of water into the cells. Although stimulation with cAMP derivates improved the formation of continuous ZO-1 and CLDN5-positive strands the shape of the cells differed markedly from those cells who did not receive the inhibitor. However, treatment with cAMP and pCPT-cAMP for 24 h seemed to decelerate the rate of cell swelling compared to the two bromine compounds. After 72 h H-89-treated cells lost their junctional integrity, intercellular gaps occurred and CLDN5 seemed to accumulate in the cytoplasm. Also cells treated with 8-Br-cAMP/Na showed increased cytosolic staining of CLDN5. However, HUVEC exposed to cAMP, pCPT-cAMP and 8-Br-cAMP still displayed continuous junction structures suggesting that expression but not necessarily membrane localization of tight junction proteins is mediated by PKA (Figure 6B).

### Effect of cAMP derivates on expression of TRIC

**Figure 7:**
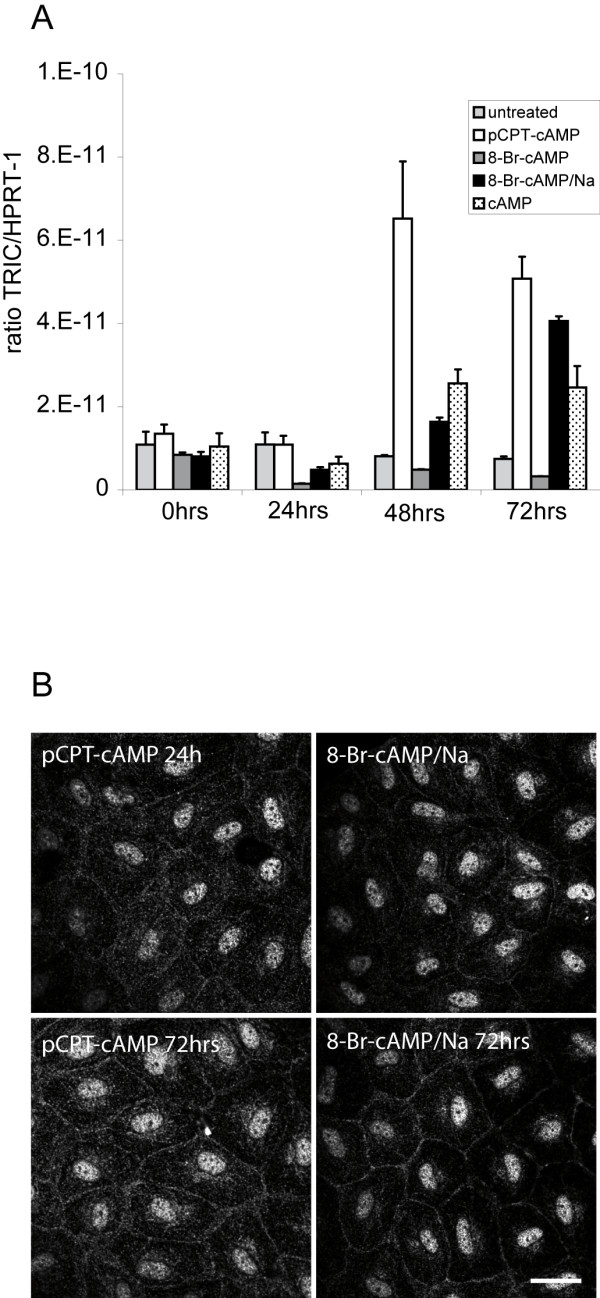
**Effect of cAMP derivates on expression and localization of TRIC**. (A) pCPT-cAMP, 8-Br-cAMP/Na and cAMP induced increased expression of TRIC mRNA as determined by real-time qPCR. Expression data were normalized to expression of HPRT-1 (n = 3). (B) pCPT-cAMP and 8-Br-cAMP/Na induced a faint membrane translocation of TRIC after 24 h. After 72 h 8-Br-cAMP/Na-stimulated cells showed rather strong membrane localization of TRIC whereas pCPT-cAMP led to only moderate TRIC staining (n = 4). The nucleus was counterstained with DAPI. Scale bar represents 25 μm.

We then analyzed expression and distribution of the recently discovered tight junction-associated protein TRIC in response to the four cAMP derivates. Real-time qPCR revealed that in HUVEC TRIC is expressed at very low level compared to expression of other junction-associated proteins like Jam-A, OCLN or CLDN5. Nevertheless, after 48 h mRNA amounts of TRIC were clearly elevated in cells stimulated with pCPT-cAMP, 8-Br-cAMP/Na or cAMP compared to untreated cells. After 72 h mRNA level of TRIC increased even further compared to untreated controls. 8-Br-cAMP, on the other hand, led to a decrease in TRIC mRNA level over the whole time period (Figure 7A).

We then performed immunostainings for TRIC. Concordant with the low expression level immunostainings for TRIC are almost not visible in untreated HUVEC (data not shown). Stimulation with pCPT-cAMP and 8-Br-cAMP/Na for 24 h induced a very faint membrane staining of TRIC whereas after 72 h TRIC localization is clearly visible along bicellular junctions (Figure 7B). However, we were not able to see TRIC membrane staining in HUVEC treated with cAMP although this compound induced an increase in TRIC mRNA expression. Of interest, different to epithelial cells TRIC could not be localized at tricellular junctions in HUVEC.

### Changes in paracellular permeability

**Figure 8:**
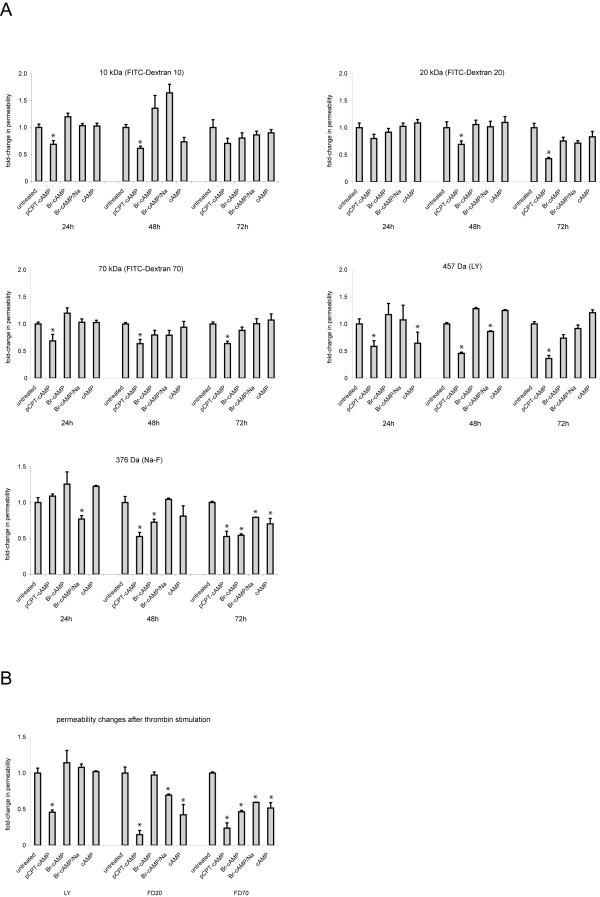
**Paracellular tracer flux assay**. (A) changes in permeability to LY, Na-F or FITC-dextrans with molecular masses of 10 kDa, 20 kDa and 70 kDa were determined after stimulation with cAMP or its derivates for 24 to 72 h. (B) Endothelial monolayer were stimulated with the different cAMP derivates for 72 h. Paracellular permeability towards LY, FD20 and FD70 was determined after pretreatment of the cells with thrombin (2 U/mL). Shown are results from three independent experiments with HUVEC derived from different donors. (*p < 0.05 compared to untreated controls).

To analyze whether the different cAMP analogues improved barrier properties in HUVEC we measured paracellular tracer flux using the small molecular weight markers Lucifer yellow (LY) and sodium-FITC (Na-F) or FITC-labeled dextrans with molecular masses of 10 to 70 kDa. All four cAMP derivates tested here led to a decrease in paracellular permeability towards Na-F after 72 h. In contrast, only pCPT-cAMP showed a beneficial effect towards flux of LY and the different FITC-dextrans at all time points (Figure 8A). In a second approach the cells were pretreated with recombinant human thrombin (2 U/mL) to test the effect of the cAMP derivates in response to a known opener of tight junctions. Interestingly, all four compounds reduced the thrombin-induced increase in permeability towards the 70 kDa dextran significantly as compared to cells that did not receive cAMP. Regarding flux of the 20 kDa dextran all but 8-Br-cAMP revealed beneficial effects on thrombin-induced permeability. In contrast, only pCPT-cAMP significantly reduced paracellular flux of the small molecular weight marker LY (Figure 8B).

## Discussion

The aim of the present study was to evaluate whether primary macrovascular endothelial cells isolated from human umbilical cords prove useful as a cell culture model to study tight junction assembly. The decision to choose HUVEC for these assays was based on the fact that umbilical cords are easy to obtain and the subsequent isolation of the endothelial cells out of the vein is well described and does not require specific technical skills [21,23]. We also decided to concentrate on human cells as a broad variety of appropriate antibodies directed against human tight and adherens junction-associated proteins are available.

Although endothelial cells apparently form dense monolayer *in vitro *microscopic analysis revealed intercellular gaps and clefts as well as discontinuous junction strands [24] demonstrating that single monocultures of endothelial cells are not sufficient to induce proper cellular junction formation. Therefore, many approaches were undertaken to overcome these limitations by co-cultivating endothelial cells with "helper cells" like astrocytes or pericytes or with their appropriate conditioned cell culture medium. Under these conditions cerebral microvascular endothelial cells succeeded in building blood-brain barrier like monolayer with enhanced transelectrical resistance and decreased paracellular permeability [15,25-27].

Another approach takes advantage of the junction-protecting effect of the second messenger cAMP. Beneath its ability to modulate PKA activity cAMP directly influences Epac, a guanine nucleotide exchange factor for Rap1 that modulates actin reorganization and distribution of adherens and tight junction-associated proteins [28,29].

We here focused on the cAMP-approach and described the effects of different cAMP analogues on formation of cellular junctions and changes in paracellular permeability in HUVEC. Although the effect of cAMP on assembly of tight junctions in endothelial cells is well known a characterization of the cellular and molecular events that are modulated by different cAMP derivates is necessary as endothelial cells from macro- or microvascular beds may differ in their response to cAMP. Moreover, evidence exists that different chemically modified cAMP analogues modulate specific cellular responses. Recently, a study by Sand and colleagues demonstrated that 8-CPT-conjugated but not bromine-conjugated cAMP analogues act as competitive thromboxane receptor antagonists [30].

We here demonstrated that, although all cAMP derivates improved formation of continuous junction strands in HUVEC, they differ regarding their cell compatibility and kinetics of junction assembly. cAMP, 8-Br-cAMP and its sodium salt 8-Br-cAMP/Na induced formation of continuous tight junction strands as soon as 24 h after addition. Nevertheless, longer incubation periods led to a disassembly of the junction strands suggesting that these compounds proved useful for solely short-termed experiments. Moreover, they did not show any significant effect on improving paracellular permeability of HUVEC monolayer and, more importantly, cAMP and 8-Br-cAMP seemed to induce extensive vacuolization when exposed for longer time periods. pCPT-cAMP, on the other hand, displayed its best effects on HUVEC when added for longer time periods (≥72h) and generated the most robust phenotype as shown by calcium switch experiments. Moreover, pCPT-cAMP is the only compound that actually induced an improvement in barrier properties. Nevertheless, all four compounds reduced thrombin-induced increases in paracellular permeability to a certain degree and partly diminished breakdown of junctions in response to calcium depletion. These results suggest that cAMP and its derivates exhibit rather protective properties towards barrier breakdown than improving existing barrier properties.

For us, it was interesting to note that the compounds tested here had different effects on transcript expression of tight junction-associated proteins in HUVEC. All four cAMP derivates induced enhanced synthesis of CLDN5 mRNA after 24 h but only in pCPT-cAMP and 8-Br-cAMP/Na-treated cells mRNA level of CLDN5 remained elevated in the next 48 h. CLDN5 is one of the main endothelial tight junction proteins and its expression correlates with formation of tight junctions and endothelial barrier properties [31-33]. On the other hand, substances known to disrupt endothelial barrier properties down-regulate CLDN5 transcript amounts [34]. Concordantly, the expression profiles of CLDN5 mRNA correlated quite well with the assembly of proper tight junction strands in HUVEC treated with the different cAMP derivates. Expression of OCLN mRNA followed a slightly different pattern. Although pCPT-cAMP and 8-Br-cAMP/Na showed the greatest effect on induction of OCLN after 24 h at the end of the experiment mRNA level did not differ compared to untreated cells. Surprisingly, the remaining two cAMP derivates did not induce any significant changes in OCLN expression. We therefore performed western blots for CLDN5 and OCLN. Whereas the protein and mRNA data for CLDN were consistent expression of OCLN was increased after stimulation with all four cAMP derivates. This discrepancy might be explained by the fact that changes in mRNA expression do not necessarily induce altered protein synthesis and that in case of doubt the protein analysis provided the most reliable data.

It is well known that cAMP exerts both PKA-dependent and -independent effects. In HUVEC cAMP-induced expression of OCLN and CLDN5 is controlled by PKA whereas membrane translocation and formation of continuous strands seemed to be independent of PKA at least for pCPT-cAMP, cAMP and 8-Br-cAMP. Interestingly, these findings are in opposite to a study by Ishizaki et al. who showed that induction of CLDN5 by pCPT-cAMP in brain microvascular endothelial cells is independent of PKA [33]. This discrepancy may be explained by the different sources of the endothelial cells; we used human macrovascular cells whereas the group of Ishizaki worked on microvascular cells isolated from porcine brain capillaries.

The intention of this study was to evaluate whether HUVEC proved to be suitable for studying endothelial tight junction formation. These primary endothelial cells express most if not all of the known endothelial adherens and tight junction-associated proteins and form proper junction strands when cultured under appropriate conditions. Nevertheless, they definitely miss some of the typical vascular endothelial characteristics like increased transelectrical resistance or highly impermeable paracellular barriers found in other endothelial cell culture models established to study e.g. blood-brain barrier properties *in vitro *[35,36]. Certainly, these different properties of the cells are due to the specific vascular beds they derived from. Whereas most of the culture models used for permeability and drug transport studies utilizes cerebral microvascular endothelial cells we worked with macrovascular venous endothelial cells from the umbilical cord. Accordingly, HUVEC are definitely not an adequate model to study blood-brain barrier-related topics. But on the other hand, umbilical cords are easy to obtain, and isolation of HUVEC does not require highly specific technical skills and is not as time-consuming as, for example, isolation of microvascular endothelial cells. Therefore, they represent a suitable culture model for studying formation or disassembly of endothelial intercellular junctions and the signaling pathways that are linked to this process.

## Conclusions

In summary, we demonstrated that for short-term experiments 8-Br-cAMP/Na and for long-term studies pCPT-cAMP have shown to be useful for formation of dense endothelial monolayer and induction of continuous tight junction strands in HUVEC. We concluded that HUVEC proved useful for studying formation and disassembly of endothelial junction architecture or characterization of junction-associated proteins. Nevertheless, their use for studying functional parameters e.g. paracellular transport or permeability seemed to be restricted under the conditions tested here.

## Methods

### Cells

HUVEC were isolated from umbilical cords as described earlier [37] with the only modification that collagenase II (Biochrom, Berlin, Germany) was used instead of chymotrypsin. Cells were maintained in EBM-2 basal medium supplemented with the EGM-2 SingleQuot kit containing growth factors, gentamycin, amphotericin-B and 2% fetal bovine serum (Lonza, Vervier, Belgium). Contaminating fibroblasts were removed using CD90-coated paramagnetic beads (Invitrogen, Karlsruhe, Germany). For all experiments HUVEC were used up to passage three. The use of HUVEC was approved by the Hannover Medical School Ethics Committee and conducted in accordance with the Declaration of Helsinki. Written informed consent was obtained from all patients.

### Stimulation of HUVEC with cAMP or cAMP-derivates

HUVEC were seeded at a density of 10^5 ^cells/ml and cultivated for 3 to 5 h to allow adherence of cells. Non-adhered cells were removed and cells were stimulated with cAMP or cAMP-derivates (Table 1) at a final concentration of 0.25 mg/ml (Sigma-Aldrich, Munich, Germany) for the indicated time intervals. Medium was changed every 24 h and HUVEC were processed according to the appropriate downstream applications. For inhibition of PKA HUVEC were pretreated for 30 minutes with the specific inhibitor H-89 (Cell Signaling/NEBiolabs, Frankfurt, Germany) at a final concentration of 10 μmol/l.

**Table 1:** cAMP derivates used in this study and their final working concentration

Substances	Abbr.	Concentration
cyclic 3',5'-adenosine monophosphate	(cAMP)	0.25 mg/ml
8-bromoadenosine 3'5'-cyclic monophosphate	(8-Br-cAMP)	0.25 mg/ml
8-bromoadenosine 3',5'-cyclic monophosphate sodium salt	(8-Br-cAMP/Na)	0.25 mg/ml
8-(4-chlorophenylthio)adenosine 3',5'-cyclic monophosphate sodium salt	(pCPT-cAMP)	0.25 mg/ml

### Determination of cellular cytotoxicity or apoptosis

Cellular cytotoxicity and metabolic activity of HUVEC due to the different cAMP derivates was determined using the CytoTox-Glo Cytotoxicity and the CellTiter-Glo Luminescent Cell Viability assay (Promega, Mannheim, Germany). Activation of caspases 3 and 7 was measured using the Caspase-Glo 3/7 assay (Promega). Assays were performed according to the manufacturer's instruction.

### Calcium switch experiments

HUVEC were normally cultivated in EGM medium that contain several sources of calcium like D-calcium pantothenate, folinic acid calcium salt or calcium chloride dehydrate. To generate a calcium-free environment the cells were cultivated for 2 h in EGM medium in the presence of EDTA (3 μM) to chelate the free calcium or in PBS without any calcium but supplemented with all growth factors contained in the Lonza single quots. After 2 h medium was changed to normal EGM medium with the appropriate cAMP derivates and cells were cultivated for another 4 h before they were fixed.

### RNA extraction and real-time quantitative PCR (qPCR)

Cells were lysed and RNA was extracted using RNeasy mini columns with an on-column DNase-digest (Qiagen, Hilden, Germany). Concentration of RNA was determined and 2 μg of total RNA was reverse transcribed using a MMLV RNase H minus point mutant reverse transcriptase (Promega). Real-time qPCR was carried out on a LightCycer480-II System (Roche Diagnostics, Penzberg, Germany) using FastStart Polymerase (Roche Diagnostics) and SYBR-Green I (Invitrogen). Gene-specific oligonucleotides for OCLN, CLDN5, Jam-A, ZO-1 and VE-cadherin were obtained from Qiagen (QuantiTect Primer Assay) with the corresponding ordering numbers listed in Table 2. PCR results were normalized to the expression of hypoxanthine guanine phosphoribosyltransferse 1 (HPRT-1) and analyzed using qGene software [38]. The sequences of the HPRT-1 oligonucleotides were TGACACTGGCAAAACAATGCA and GGTCCTTTTCACCAGCAAGCT. Each experiment was repeated at least three times with HUVEC derived from different donors.

**Table 2:** Ordering information of the QuantiTect primer assays

Oligonucleotides	Ordering numbers (Qiagen)
Jam-A	QT00083972
CLDN5	QT01681232
OCLN	QT00081844
TRIC	QT01031919
VE-cadherin	QT00013244
ZO-1	QT00077308

### Western blot

Cells were lysed in ice-cold 1 × lysis buffer (NEBiolabs, Frankfurt, Germany) containing protease and phosphatase inhibitor cocktail tablets (Roche Diagnostics). Protein content of the samples was determined using the BCA protein assay kit (Thermo Fisher Scientific, Bonn, Germany). 50 μg of whole cell lysates was separated by SDS PAGE electrophoresis and blotted on a PVDF nylon membrane. Filters were incubated with the appropriate primary antibody followed by incubation with a HRP-conjugated secondary antibody. The bands were visualized by Western Lighting chemiluminiscence reagent (Perkin Elmer, Rodgau, Germany) and quantified by densitometry using a CCD camera and Quantity One software (Biorad Laboratories, Munich, Germany).

### Immunocytochemistry

Cells were grown on collagen-coated glass cover slips and fixed in ice-cold acetone for 15 minutes at -20°C followed by permeabilization with ice-cold methanol for 20 min at -20°C. After washing with PBS the cells were blocked in normal donkey serum, incubated with the primary antibody for one hour followed by incubation with the appropriate secondary antibody coupled to ALEXA-488 or ALEXA-546 (Invitrogen) for an additional hour. DNA was counterstained with DAPI (Sigma Aldrich) and confocal images were taken using a Leica DM IRB microscope with a TCS SP3 AOBS scan head equipped with argon and krypton laser beams. Micrographs were obtained using a HCX PL APO 63 × 1.4 numerical aperture objective. Antibodies used in this study were a murine monoclonal anti-VE-cadherin (clone 55-7H1 from BD Pharmingen, Heidelberg, Germany; working concentration 1 ng/μl), a murine monoclonal anti-ZO-1 antibody (clone 1/ZO-1 from BD Pharmingen, working concentration 2.5 ng/μl), a murine monoclonal anti-OCLN antibody (clone 3F10 from Invitrogen, working concentration 5 ng/μL), a polyclonal goat anti-Jam-A antibody (R&D Systems, Wiesbaden, Germany, working concentration 1 ng/μl), a polyclonal anti-TRIC antibody (Invitrogen) and a rabbit anti-CLDN5 antibody (Santa Cruz Biotechniques, Heidelberg, Germany, working concentration 2 ng/μl).

### Paracellular tracer flux assay

For determination of paracellular permeability HUVEC were seeded into the upper compartment of transwell devices with a pore size of 0.4 μm and cultivated for two days to reach confluence. After stimulation for 24 to 72 h with cAMP or its derivates the low molecular weight markers Lucifer yellow (LY, 457 Da, 20 μmol/L) and sodium fluorescein (Na-F, 10 μg/mL) or fluorescein (FITC)-coupled dextrans with the molecular masses of 10 kDa, 20 kDa or 70 kDa (of 200 μg/ml) were added to the upper compartment (Sigma-Alrich). In some experiments cells were pretreated with thrombin (2 U/mL, Merck, Darmstadt, Germany). After 30 minutes the concentrations of FITC-labeled dextrans in the lower compartment were measured using an ELISA reader equipped for fluorescence measurement (Tecan, Crailsheim, Germany).

### Statistical analysis

Results were expressed as the mean ± SD of at least three independent experiments with cells derived from different donors. Statistical significance was calculated using Mann-Whitney-U test.

## Authors' contributions

MB performed experiments, analyzed the data and drafted the manuscript, KW performed the cell culture, MH analyzed the data, participated in the design of the study and approved the final manuscript, TK designed and coordinated the study, performed the confocal microscopy analysis and wrote the manuscript. All authors read and approved the final manuscript.

## Supplementary Material

Additional file 1 **Immunostainings of VE-cadherin**. HUVEC were stimulated with cAMP or different cAMP derivates for 24 to 72 h and stained for VE-cadherin. The nucleus was counterstained with DAPI. Shown are representative confocal images of at least three independent experiments with HUVEC derived from different donors. Scale bar represents 25 μm. Click here for file

Additional file 2 **Immunostainings of tight junction marker after 48 h stimulation with cAMP derivates**. Shown are representative micrographs of immunostainings for ZO-1, OCLN, Jam-A and CLDN5 in HUVEC that were stimulated with cAMP or its derivates for 48 h. The nucleus was counterstained with DAPI. Scale bar represents 25 μm. Click here for file

Additional file 3 **mRNA expression data of junction-associated proteins**. HUVEC were stimulated for 24 to 72 h with cAMP or its derivates. RNA was extracted, reverse transcribed and expression of VE-cadherin, ZO-1 and Jam-A was determined by real-time qPCR. Expression data were normalized to relative transcript amounts of HPRT-1 (n = 3). Click here for file

Additional file 4 **cAMP-induced expression of CLDN5 and OCLN is mediated by PKA**. (A) HUVEC were pretreated with H-89 for 30 minutes and stimulated with cAMP or its derivates for up to 72 h. mRNA expression of OCLN and CLDN5 were determined by real-time qPCR and normalized to expression of HPRT-1 (n = 3). (B) Shown are representative western blot images of HUVEC pretreated with H-89 for 30 minutes and stimulated with cAMP derivates for up to 72 h. Protein amounts of tubulin served as loading control (n = 2). Click here for file
