# Supplementary figures and images for: Effect of cAMP derivates on assembly and maintenance of tight junctions in human umbilical vein endothelial cells
Source: BMC Cell Biol. 2010 Sep 7;11:68. doi: 10.1186/1471-2121-11-68 (PMC2941681; doi:10.1186/1471-2121-11-68)

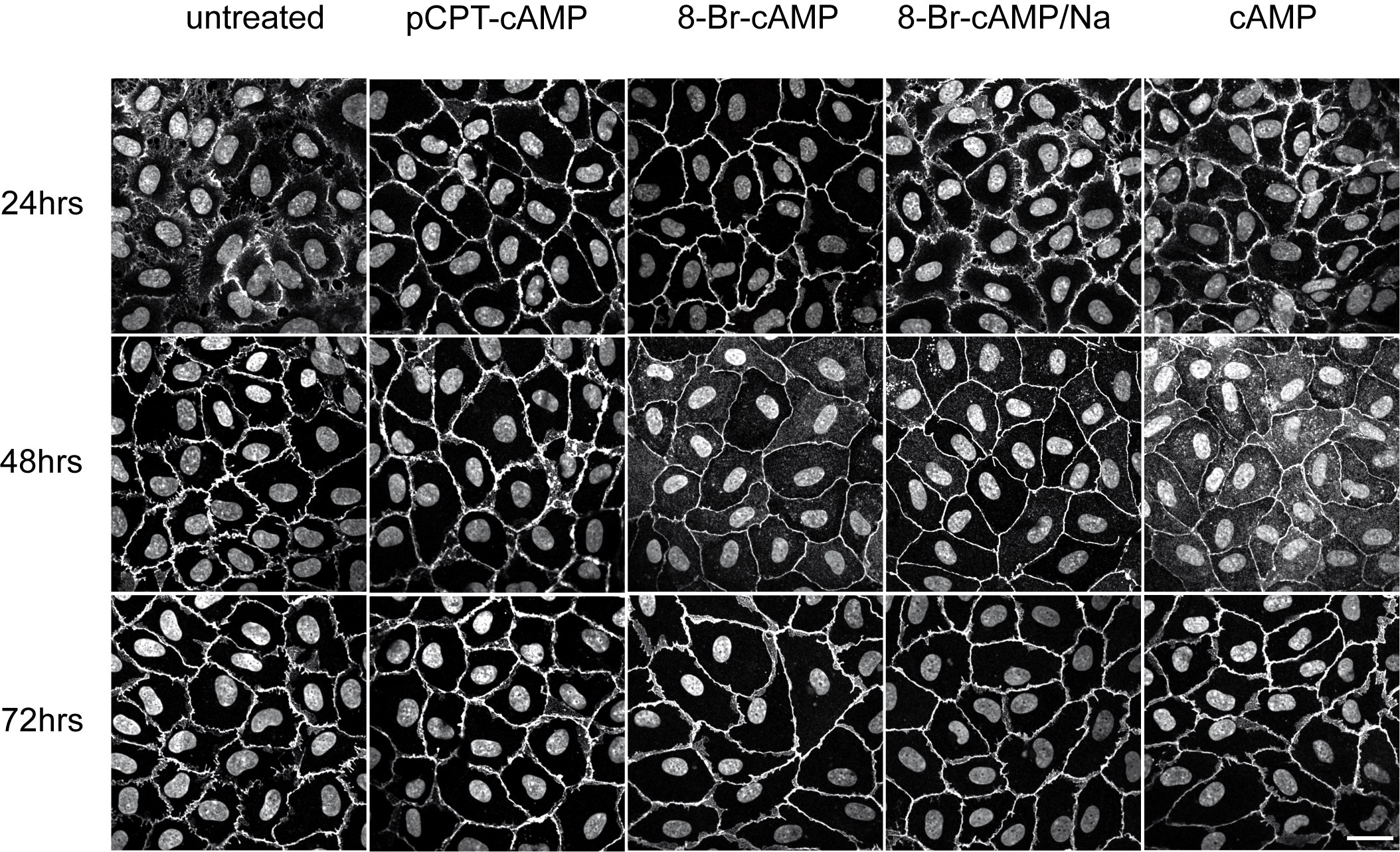

Supplement: Additional file 1 — Immunostainings of VE-cadherin. HUVEC were stimulated with cAMP or different cAMP derivates for 24 to 72 h and stained for VE-cadherin. The nucleus was counterstained with DAPI. Shown are representative confocal images of at least three independent experiments with HUVEC derived from different donors. Scale bar represents 25 μm. [file 1471-2121-11-68-S1.PNG]

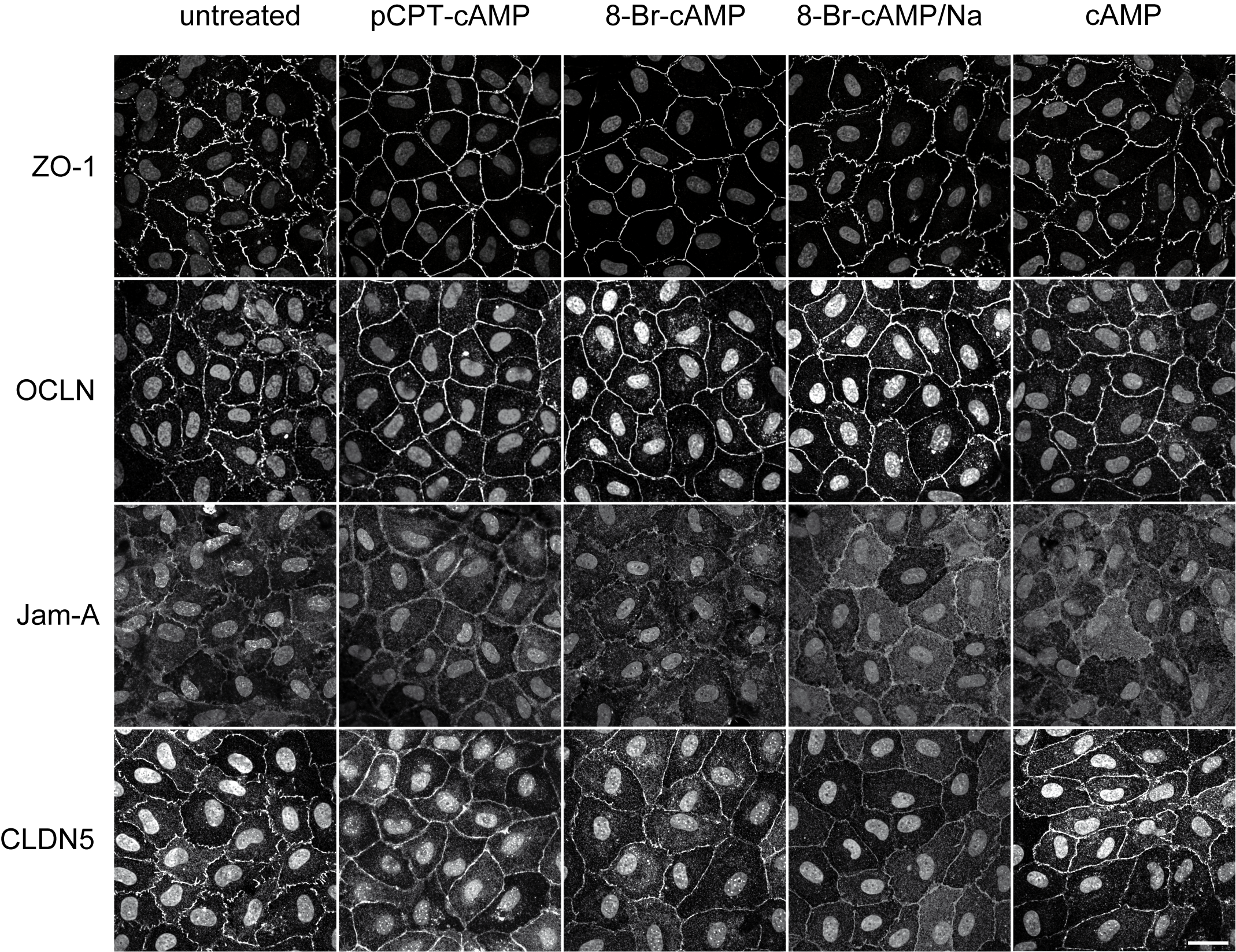

Supplement: Additional file 2 — Immunostainings of tight junction marker after 48 h stimulation with cAMP derivates. Shown are representative micrographs of immunostainings for ZO-1, OCLN, Jam-A and CLDN5 in HUVEC that were stimulated with cAMP or its derivates for 48 h. The nucleus was counterstained with DAPI. Scale bar represents 25 μm. [file 1471-2121-11-68-S2.PNG]
